# Breast Tumor Angiogenesis and Tumor-Associated Macrophages: Histopathologist's Perspective

**DOI:** 10.4061/2011/572706

**Published:** 2011-06-15

**Authors:** Ewe Seng Ch'ng, Hasnan Jaafar, Sharifah Emilia Tuan Sharif

**Affiliations:** Department of Pathology, School of Medical Sciences, Universiti Sains Malaysia, 16150 Kubang Kerian, Kelantan, Malaysia

## Abstract

Much progress has been made since the conceptualization of tumor angiogenesis—the induction of growth of new blood vessels by tumor—as a salient feature of clinically significant primary or metastatic cancers. From a practicing histopathologist's point of view, we appraise the application of this concept in breast cancer with particular reference to the evaluation of proangiogenic factors and the assessment of new microvessels in histopathological examination. Recently, much focus has also been centered on the active roles played by tumor-associated macrophages in relation to tumor angiogenesis. We review the literature; many data supporting this facet of tumor angiogenesis were derived from the breast cancer models. We scrutinize the large body of clinical evidence exploring the link between the tumor-associated macrophages and breast tumor angiogenesis and discuss particularly the methodology and limitations of incorporating such an assessment in histopathological examination.

## 1. Introduction

Angiogenesis, the growth and remodeling of new blood vessels, is one of the hallmarks of cancer. Acquiring proangiogenic phenotype, tumor cells produce and release proangiogenic factors to initiate angiogenesis whereby the ensuing tumor growth, invasion, and metastasis take place. Subject to this angiogenic switch tenet for its progression, breast cancer has been shown to produce a number of proangiogenic factors. Studies have demonstrated that the evaluation of these proangiogenic factors carries predictive and prognostic values [1, 2]. Prognostic significance of tumor angiogenesis has also been highlighted in clinical studies where higher microvessel densities correlate with poorer survival outcome [3]. Via the control of angiogenesis, another dimension in therapeutic intervention is now unfolded. 

In relation to tumor angiogenesis, recent research also focuses on the role of tumor microenvironment. Tumor-associated macrophages, a major component in the leukocytic infiltration in tumor, have aroused much research interest since the propositions of their active involvement in tumor progression [4, 5]. Best summarized as M2 phenotype, tumor-associated macrophages show anti-inflammatory and tumor-promoting characteristics, especially in relation to tumor angiogenesis. Apart from the *in vitro* and *in vivo* animal studies based on the breast cancer models, there is accumulating evidence from the clinical studies that suggests tumor-promoting features of tumor-associated macrophages in breast cancer [6, 7].

In this paper, we outline the conceptual development of breast tumor angiogenesis and evaluate the methodology and limitations of quantifying proangiogenic factors and microvessel density in the assessment of tumor angiogenesis in breast cancer. We summarize the pertinent experimental and clinical data exploring the link between the tumor-associated macrophages and breast tumor angiogenesis, emphasizing the methodology and limitations of histopathological assessment in this regard.

## 2. Breast Cancer and Tumor Angiogenesis

### 2.1. Tumor Angiogenesis Is One of the Hallmarks of Cancer

Cancer development and progression is a complex multistep process where novel capabilities, the hallmarks of cancer, are acquired through the accumulation of multiple genetic alternations. These hallmarks of cancer include not only the tumor's cellular autonomy such as self-sufficiency in growth signals and limitless replicative potential but also the abilities to interact with the surrounding stroma such as development of sustained angiogenesis [8]. In particular, the ability to activate angiogenesis plays a crucial role in controlling tumor progression because tumor growth, invasion, and metastasis are angiogenesis dependent [9].

Folkman first proposed angiogenesis dependency of tumor growth and metastasis in 1971. He hypothesized that tumor would remain in dormancy at a microscopic size (1-2 millimeter) in the absence of angiogenesis [10]. This is comprehensible because a tumor, similar to its nonneoplastic counterpart, requires adequate supply of oxygen and nutrients and an effective means to dispose its waste products for survival and growth; these metabolic needs can be fulfilled through tumor-induced angiogenesis [11]. In fact, all mammalian cells including the tumor cells are restricted to within 100–200 *μ*m of a capillary blood vessel due to oxygen diffusion limit of about 100 *μ*m [12].

In addition, angiogenesis facilitates metastasis. The newly formed tumor blood vessels are structurally abnormal. For instance, increased numbers of fenestrations, vesicles, and vesicovacuolar channels and a lack of normal basement membrane are common in tumor vessels [13]. These abnormal blood vessels are consequently more permeative and would constitute the easier entry point for tumor cells to enter into the circulation and hence distant micrometastases [14]. The ensuing micrometastases at ectopic places would remain dormant unless secondary angiogenesis occurs and paves the way for the establishment of a clinically evident disease [15]. 

Angiogenic switch concept has been postulated to explain the mechanism underlying tumor dependence on angiogenesis to escape from dormancy. Under this concept, the balance of proangiogenic and antiangiogenic factors would ultimately determine the activation status of the switch. When the balance is tilted towards the angiogenic end, the angiogenic switch is turned on; transition from the avascular phase into the vascular phase will be triggered, permitting exponential tumor growth and subsequent transformation into an aggressive phenotype [16]. 

Factors regulating this angiogenic switch have been extensively explored across various tumors. More than 40 endogenous proangiogenic and antiangiogenic factors are now known [17].

### 2.2. Immunohistochemical Evaluation of Proangiogenic Factors Produced by Breast Cancer Needs Validation

Subject to the angiogenic switch tenet for its progression, angiogenesis has been shown by studies to be initiated in the hyperplastic state and to intensify towards the invasive carcinoma end of spectrum in breast cancer [18–20]. Breast cancer has been shown to express at least six different proangiogenic factors. These include vascular endothelial growth factor (VEGF) and its four isoforms (121, 165, 189, and 206 amino acids), transforming growth factor (TGF)-*β*1, pleiotrophin, acidic and basic fibroblast growth factor (FGF), placental growth factor, and thymidine phosphorylase (platelet-derived endothelial cell growth factor) [21]. 

Among these factors, VEGF and associated factors have been the centre of many studies addressing the clinical significance of proangiogenic factors in terms of predictive and prognostic values. In predicting the response to chemotherapy or tamoxifen, higher level of VEGF in tumor by immunohistochemistry or in tumor cytosol by EIA/ELISA method forecasted poorer response in a number of studies [1]. In a review of breast cancer patients, an overwhelming 81% of 37 clinical studies demonstrated that the VEGF level in tumor or serum, as determined by either immunohistochemistry or ELISA method, serves as an adverse prognostic marker for disease-free or overall survival [2]. 

Selected studies within the last 10 years exploring the prognostic significance of VEGF and associated factors in breast cancers using immunohistochemistry methodology are highlighted in Table 1, considering immunohistochemistry as a part of routine histopathological examination. As shown in Table 1, many recent studies performing immunohistochemistry in evaluating the expression of VEGF and associated factors failed to demonstrate the prognostic values of these factors in terms of disease-free survival or overall survival [22–37]. Some studies showed that these proangiogenic factors act as a poor prognostic marker but lose their significance in multivariate analysis [38–48]. A number of limitations in the immunohistochemistry methodology could account for these observations. Morphometric assessment is inevitably subject to the individual evaluator's subjectivity. In addition, there is no validated uniform scoring system employed in the reported studies. Primary detecting antibodies from various sources in these studies would give rise to variable detection sensitivity and specificity of the targeted proangiogenic factors. The establishment of a validated immunohistochemistry evaluation is therefore essential to gain comparable data across clinical studies. In addition, this is particularly relevant if the pathology reporting of breast cancer is to incorporate information regarding proangiogenic factors for therapeutic consideration in view of availability of antiangiogenic therapy in on-going clinical trials.

### 2.3. Methodological Inconsistency in the Assessment of Tumor Vascularity in Breast Cancer Limits Its Clinical Prognostic Value

Apart from the evaluation of the regulating factors in the angiogenic switch, the quantification of angiogenesis in breast cancer per se has its own clinical prognostic values. In a landmark paper, Weidner et al. demonstrated that by immunostaining the blood vessels, the number of microvessels per 200x field in the highest neovascularization areas (hot spots) correlated with distant metastasis in breast cancer patients, corresponding to a 1.17-fold (95% CI = 1.02, 1.34) increase in the distant metastasis risk for every increase in 10 microvessels [49]. Since then, microvessel density determined by this method and its variants has become the most popular surrogate marker in assessing angiogenesis across various cancers [50]. In breast cancer, higher microvessel densities predict higher risk of subsequent *in situ* cancers and invasive recurrence of previous *in situ* cancers [51], poorer response to treatment [52], and higher occurrence of micrometastases [53–55]. In a meta-analysis of 25 independent studies, high microvessel density significantly predicted poor relapse-free survival and overall survival (both RR = 1.54, 95% CI  =  1.29, 1.84) [3]. 

However, scrutinizing each of the studies included in the above-mentioned meta-analysis, variations in results regarding prognostic value of microvessel density in breast cancer patients' survival are apparent [3]. The choice of antibodies to highlight the blood vessels in various studies could be a contributing factor because each antibody has its own specificity and sensitivity against the endothelial cells of the blood vessels. Among the commonly used antibodies are antibodies against factor-VIII-related antigen, CD31, and CD34. Anti-CD34 is now considered the optimal marker for its higher sensitivity without high failure rate in antigen retrieval for invasive breast carcinoma studies [3, 56–58].

Another factor to consider in assessing microvessel density is the variations from the original method designed by Weidner et al. These include variables such as the number of hot spots counted, the areas and fields of magnification (magnification of a field area of 200x or 400x), the subjectivity in identification of what constitutes a stained blood vessel, and also the descriptive statistics in reporting the number of microvessel density (the mean or the highest value). To overcome the subjectivity of observers, a 25-point Chalkley microscope eyepiece graticule has been introduced. The graticule is orientated in such a way that it gives the maximum number of graticule points overlapping the highlighted vessels. This method measures relative area and has strong association with vessel area and number [3, 57–59]. Both the conventional optical assessment method and the Chalkley method have been used in studies that demonstrated increased microvessel density as a poor prognostic factor [3, 60, 61]. Controversies over the best methodology remain despite a proposed consensus of using Chalkley method in angiogenesis quantification in solid human tumors [58].

### 2.4. Antiangiogenic Therapy Gives Promising Results in Preclinical Studies but Not in Clinical Trials of Metastatic Breast Cancer

Given the pivotal roles of proangiogenic factors in tumor angiogenesis, these factors serve as reasonable pharmacological targets for inhibition of tumor angiogenesis. Among these factors, blockage of the VEGF pathways was the focus of many preclinical studies because VEGF is the most potent proangiogenic factor [62]. 

A number of experimental xenograft models using different tumor cell types including breast carcinomas showed that anti-VEGF therapy resulted in 25% to 95% of tumor growth inhibition in a dose-dependant manner. Functionally, tumor microvascular permeability was also reduced [63]. Upon antiangiogenic drug treatment, tumor vessels remodel and transiently resemble the normal vessels. During this normalization window, the normalized tumor vessels are believed to be more efficient in delivering the nutrients as well as cytotoxic drugs and oxygen, potentiating the effects of the combination of cytotoxic and antiangiogenic therapies targeting the tumor cells and endothelial cells, respectively [64, 65]. This tumor vasculature normalization model provides a rationale for the observed better effects of combined cytotoxic and anti-VEGF therapy as compared to single-agent treatment in preclinical studies [63]. Although this tumor vasculature normalization model is conceptually appealing, histologic examination of vasculature normalization in clinical setting to identify the optimized normalization window is limited in several aspects such as representative multiple small biopsies that would be hardly obtained for the global assessment of the solid tumor [64, 65].

Results from the preclinical studies pave the way for the use of anti-VEGF therapy in clinical trials. However, the results from the recent phase III clinical trials in breast cancer treatment using bevacizumab, a humanized monoclonal antibody against VEGF, are not as promising as in animal studies. A meta-analysis including five reported clinical trials involving metastatic breast cancer patients showed that the combined bevacizumab and chemotherapy arm had better objective response (RR = 1.26, 95% CI  =  1.17, 1.37) and progression-free survival (HR = 0.70, 95% CI  =  0.60, 0.82) as compared to the chemotherapy-alone arm. However, no significant advantage was seen with the addition of bevacizumab as compared to the chemotherapy-alone arm for overall survival (HR = 0.90, 95% CI  =  0.80, 1.03) [66]. 

Two trials have published results and one has a report published recently for further inspection of the study design [83–85]. Although bevacizumab specifically blocks the VEGF-mediated pathways, all of these trials used bevacizumab as a general therapy given on a population basis without considering the specific molecular phenotype of the breast cancer. VEGF expression profile of the cancers was not investigated in the enrolled patients and the best methodology of evaluation has not yet been validated. Redundancy of other proangiogenic factors might also play important roles in advanced breast cancer. Consideration in these factors is needed to better stratify the patients who will most likely benefit from the VEGF-targeted therapy.

## 3. Roles of Tumor-Associated Macrophages in Breast Cancer

### 3.1. Macrophages Are Recruited into the Tumor

Infiltration of leukocytes in tumors was first recognized by Virchow in 1863 prompting him to postulate the link between the origin of cancer and inflammation [4]. This link, arbitrarily termed the extrinsic pathway, increases the risk of cancer development, exemplified by inflammatory conditions associated with malignancy such as ulcerative colitis linked to the development of colon cancer. In contrast, it is now evident that the intrinsic pathway, genetic alterations causing cancer without casual relationship to inflammatory processes, also leads to a protumor inflammatory microenvironment [86].

Among the heterogeneous populations of the leukocytic infiltrates, it has now been established that macrophages constitute the major proportion; for instance, up to 50% of cell mass in breast carcinoma is composed of macrophages [87]. These macrophages are called tumor-associated macrophages. They are mostly derived from the peripheral blood monocytes and recruited into the tumor by a wide range of chemokines and growth factors released by the tumor cells. Among these, CC chemokines, particularly CCL2 (formally monocyte chemoattractant protein-1 or MCP-1) and CCL5, and growth factors such as colony-stimulating factor-1 (CSF-1) and vascular endothelial growth factor (VEGF) are strongly implicated in macrophage recruitment in various tumors including breast cancer [88].

### 3.2. Tumor-Associated Macrophages Are Polarized into M2 Phenotype in Tumor Microenvironment

The interaction between the tumor cells and the recruited tumor-associated macrophages has aroused much research study interest. The classical view of tumor-associated macrophages displaying antitumor response to destroy the tumor cells, similar to their pathophysiological response to microbial invasion, has however been confronted by a large number of studies that contradictorily showed their opposite protumor response. This paradoxical function of tumor-associated macrophages in relation to tumor is explained by the macrophage balance hypothesis where the outcomes of the tumor-associated macrophages depend on the polarization between two extremes of a continuum: M1 as proinflammatory and microbicidal/tumoricidal phenotype in contrast to M2 as anti-inflammatory and tumor-promoting phenotype [5].

Clinical studies across various human tumors exploring correlation between tumor-associated macrophage density and prognosis have shown constant strong inverse relationship in carcinomas of breast and cervix but a minority of conflicting results in prostate, stomach, and lung cancers [89]. These results suggest the importance of tumor microenvironment in tilting the macrophage balance and support largely the polarization of macrophage into protumor M2 phenotype by most tumors, including the breast carcinomas.

### 3.3. Tumor-Associated Macrophages Enhance Tumor Progression in Breast Cancer

*In vitro* and *in vivo* animal studies, especially the animal model of mammary tumor, have shed much light on the roles of tumor-associated macrophages in tumor progression. For instance, when a null mutation *colony stimulating factor-1* gene was crossed into transgenic mice susceptible to mammary cancer due to the expression of the polyoma middle T antigen oncogene (PyMT mice), depletion of macrophages resulted in delayed tumor progression and tumor metastasis. In contrast, overexpression of *CSF-1* gene resulted in increased macrophage infiltrates and in turn accelerated tumor progression and tumor metastasis [90]. 

Restricting the data pertinent to human breast cancer, the increased tumor-associated macrophages number correlates with high proliferative activity of the tumor cells as indicated by higher mitotic grade and Ki-67 labelling [6, 69, 76, 77, 81]. This association could be explained by the direct mitogenic stimulation of tumor cells by tumor-associated macrophages or indirect effect via stimulation of tumor angiogenesis by tumor-associated macrophages as discussed below. For the former possibility, tumor-associated macrophages indeed express and release a wide range of growth factors such as epidermal growth factor, basic fibroblast growth factor-2 (FGF-2), transforming growth factor-*β*, VEGF, and platelet-derived growth factor (PDGF) [91]. In particular, it has been shown that tumor-associated macrophages secrete epidermal growth factor, but the normal or malignant breast cancer cells do not [92]. Many breast cancers express epidermal growth factor receptor [93], which upon activation by this ligand leads to tumor survival and proliferation [94]. 

Furthermore, a paracrine loop between breast cancer cells and tumor-associated macrophages could promote the invasion of breast carcinoma via reciprocal stimulation because CSF-1 secreted by breast cancer cells recruits macrophages, and epidermal growth factor derived from the recruited macrophages promotes tumor cell motility [95]. In addition, tumor-associated macrophages produce enzymes and inhibitors, which regulate the digestion of the extracellular matrix such as matrix metalloproteinases (MMPs) [96] and urokinase-type plasminogen activator (uPA) [97]. Accordingly, degradation of the extracellular matrix by these macrophage proteases would facilitate the invasion of tumor cells into the stroma and hence metastasis [89, 98]. This constitutes one of the mechanisms explaining the association of poor prognosis in breast cancer with higher macrophage density in clinical studies [7, 71, 77, 79].

### 3.4. Tumor-Associated Macrophages Enhance Tumor Angiogenesis in Breast Cancer

As discussed above, tumor angiogenesis is crucial for tumor progression. Tumor angiogenesis was initially thought to be induced only by tumor cells themselves; however, tumor-associated macrophages are indeed a major player in the regulation of tumor angiogenesis [99]. It is now evident that tumor-associated macrophages recruited into the tumor microenvironment are producers of a wide range of proangiogenic factors, including IL-1, VEGF, IL-8, bFGF, and TNF-*α* [100]. 

The process of activation and transformation of the tumor-associated macrophages into this proangiogenic phenotype is dependant on several tumor microenvironmental stress factors such as low oxygen, low pH, and high lactate concentration [101]. Tumor hypoxia appears to be the major regulating factor. One study has shown that the median pO2 value in breast cancer was 30 mmHg compared to 65 mmHg in normal tissue and could be as low as between zero and 2.5 mmHg [102]. Macrophages are attracted to these hypoxic areas [70, 73], and via the hypoxia-induced pathway, large numbers of genes encoding the proangiogenic factors are dramatically upregulated in the tumor-associated macrophages [103]. 

The first clinical study correlating tumor-associated macrophages and angiogenesis also came from a study on breast cancer. Significant correlation between the two was shown in addition to the prognostic value of tumor-associated macrophages, implying the crucial role of angiogenesis driven by tumor-associated macrophages in breast cancer progression [7]. Later clinical studies also produced similar findings [76, 77, 79, 81]. *In vivo* animal study employing PyMT mice showed that the inhibition of macrophage maturation and infiltration into tumors delayed angiogenesis and tumor progression, providing evidence of causal role of tumor-associated macrophages in tumor angiogenesis [104].

### 3.5. Assessment of Tumor-Associated Macrophages in Breast Cancer by Immunohistochemistry Varies in Clinical Studies

Major findings in recent clinical studies exploring the link between the tumor-associated macrophages and other clinicopathological parameters in invasive breast carcinomas using immunohistochemistry are summarized in Table 2. These studies are generally agreeable in terms of association between the density of macrophages and clinicopathological parameters related to tumor progression. Besides, the significant association between density of macrophages and microvessel density implies the role of tumor-associated macrophages in tumor angiogenesis.

As shown in Table 2, in all but two studies, the antibody against CD68 was used to highlight the presence of macrophages. However, there is variation in the methods used to assess tumor-associated macrophages in these studies. Some studies used semiquantitative methods [68, 76, 78, 79] and others used quantitative methods with variation in selection of fields and count [6, 7, 70–75, 77, 80–82]. These variations in assessment method would give rise to minor discrepancies among the studies. In particular, no much attention was given to the location of tumor-associated macrophages in relation to breast carcinomas. It is known that the tumor-associated macrophages are attracted to hypoxic tumor areas, and angiogenesis is likely to be induced at these hypoxic areas. Most studies used the “hot spot” method to identify the areas of the highest number of tumor-associated macrophages [7, 70–75, 77, 80–82]. These studies most probably have evaluated the tumor-associated macrophages at tumor margin where angiogenesis occurs, as opposed to tumor-associated macrophages within the tumor nest where information regarding their role is still lacking [105]. Evaluation by this “hot spot” methodology could also alleviate the concern about the confounding macrophages induced by biopsies prior to surgical resection of the tumor, as it is unlikely that a biopsy tract would induce accumulation of macrophages only at the tumor margin. 

Given the many positive findings regarding the association of macrophages and breast tumor progression, a standardized evaluation method for assessing tumor-associated macrophages is therefore necessary to harmonize future research. A consensus of using “hot spot” method with particular reference to tumor-associated macrophages in tumor stroma would probably constitute such a template for examination.

### 3.6. Targeting Tumor-Associated Macrophages in Breast Cancer Represents an Attractive Approach

A plethora of growth factors, cytokines, and chemokines are employed in the process of recruitment, survival, activation and polarization, proangiogenic activity, and matrix remodeling of tumor-associated macrophages. These factors represent reasonable therapeutic targets [106]. For instance, in an experimental breast cancer model, antagonizing the chemokine CCL5 receptors expressed on the macrophages reduced the number of tumor-associated macrophages and slowed the tumor growth [107]. Using the anti-VEGF antibody to treat breast cancer xenografts, in addition to the inhibition of angiogenesis, infiltration of tumor-associated macrophages was also reduced. In these studies, tumor growth and distant metastases were inhibited [108, 109]. Although the contribution of reduction of tumor-associated macrophages to the observed results in these experimental studies has yet to be determined, pathological correlation in this aspect in the clinical trials employing anti-VEGF therapy would be of great interest.

## 4. Conclusion

In conclusion, the salient points regarding trilateral relationship among breast cancer cells, tumor-associated macrophages, and tumor angiogenesis are

1. breast cancer progression is dependent on tumor angiogenesis,
2. breast cancer cells are able to regulate tumor angiogenesis via production of proangiogenic factors,
3. tumor-associated macrophages have emerged as a major player in regulating breast cancer progression,
4. as a major regulatory mechanism in tumor progression, tumor-associated macrophages enhance breast tumor angiogenesis,
5. breast cancer progression involves reciprocal interactions between breast cancer cells and tumor-associated macrophages.

At the tissue level, the assessment of the relationship between these three compartments is feasible by histopathological examination coupled with immunohistochemistry. Despite its limitations, microvessel density has been widely used as a surrogate marker for tumor angiogenesis. Establishment of a validated immunohistochemical evaluation of proangiogenic factors produced by breast cancer is essential. Information regarding expression profile of proangiogenic factors might help to stratify patients receiving antiangiogenic therapy. Tumor-associated macrophage density can be graded in similar manner as microvessel density evaluation. Assessment in this regard would possibly constitute another important item in histopathological examination for prognostication, considering therapeutic advances targeting tumor-associated macrophages.

##  Conflict of Interests

All authors have no conflict of interests.

## Figures and Tables

**Table 1:** Summary of the selected studies in the last 10 years exploring the prognostic significance of VEGF and associated factors using immunohistochemistry in breast cancers.

Patients	Assessment of VEGF expression	Prognostic value of VEGF expression
98 stage II ductal breast cancers [26]	Antibody: monoclonal anti-VEGF165 Scoring system: 0 = none, 1 = <33%, 2 = 33–66%, 3 = >66% positive tumor cells	VEGF had no prognostic significance for overall survival or disease-free survival

48 triple negative breast cancers not receiving systemic adjuvant treatment from 500 primary breast cancers using tissue microarrays [24]	Antibody: polyclonal anti-VEGF Scoring system: cytoplasmic staining intensity was scored from 0 to 3 High expression had score 3	VEGF had no prognostic significance for 5-year breast-cancer-specific survival

125 stage II node-positive invasive ductal carcinomas, NOS 25 stage II node-positive invasive lobular carcinomas [23]	Antibody: polyclonal anti-VEGF-C Staining was graded as strong, medium, or weak-to-absent expression	VEGF-C had no prognostic significance for overall survival or disease-free survival

172 primary breast cancer [25]	Antibody: anti-VEGF-A Scoring system: staining intensity was graded from 0 (negative) to 3 (strong intensity) Positive cases had score 1–3	VEGF-A had no prognostic significance for recurrence-free survival

116 invasive ductal breast cancers [27]	Antibody: anti-VEGF Scoring system: positive cases had >10% positive tumor cell staining	VEGF-A had no prognostic significance for overall survival in multivariate analysis

52 infiltrating ductal carcinomas, 4 intraductal carcinomas, 3 mucinous adenocarcinomas,1 medullary carcinoma, 1 inflammatory breast carcinoma [38]	Antibody: anti-VEGF-C, anti-VEGF-D Scoring system: sum of staining intensity (0 = negative to 3 = strong) and percentage of positive cells (0 = 0%, 1 = 1–10%, 2 = 11–30%, 3 = 31–50%, 4 = 51–100%) High-expression group had score 4–7	High expression of VEGF-C/D had poorer disease-free survival and overall survival

59 invasive ductal carcinomas, NOS 11 other types of invasive breast cancer [39]	Antibody: polyclonal anti-VEGF-C Scoring system: negative, 1+ (focal expression in <5%), 2+ (focal expression in 5–20%), 3+ (diffuse expression in >20%) High-expression group had score above 2+	Shorter disease-free survival and overall survival for high expression of VEGF-C in univariate analysis

215 high-risk primary breast cancers with extensive axillary involvement [28]	Antibody: monoclonal anti-VEGF Staining intensity was graded from 0 to 3+ Positive cases are those having any tumor areas with positive staining	VEGF had no prognostic significance for overall survival or relapse-free survival

177 invasive breast cancers [40]	Antibody: monoclonal anti-VEGF-A, anti-VEGF-D, polyclonal anti-VEGF-C Scoring system: H score (multiplying percentage of positive carcinoma cells by the staining intensity graded 0 to 3) High-expressing tumors had score above the median score	(1) Shorter overall survival for high expression of VEGF-A in univariate analysis (2) Shorter overall survival and disease-free interval for high expression of VEGF-C in univariate and multivariate analyses (3) No prognostic significance for VEGF-D (4) Tumours with high expression of both VEGF-A and -C had significantly shorter overall survival

130 invasive ductal carcinomas, 30 invasive lobular carcinomas [41]	Antibody: polyclonal anti-VEGF-B, monoclonal anti-VEGF-A (165, 189, 206 a.a.) Scoring system: 0 (no or weak staining in <10%), 1 (weak-to-moderate staining in 11–20%), 2 (moderate-to-strong staining 21–50%), 3 (strong staining in >50%) Positive cases had score above 2	(1) VEGF-A had no prognostic significance (2) Unfavorable disease-free and overall survival for VEGF-B-positive cases in lymph node metastases cases

136 invasive ductal carcinomas, 31 invasive lobular carcinomas [42]	Antibody: polyclonal anti-VEGF-C, polyclonal anti-VEGF-D Scoring system: positive cases had at least 10% immunoreactive tumor cells	Poorer overall survival for VEGF-C-positive cases VEGF-D had no prognostic significance
80 invasive ductal carcinomas, 15 ductal carcinomas *in situ*, 5 lobular carcinomas *in situ*, 14 invasive lobular carcinomas, 6 medullary carcinomas, 2 tubular carcinomas [43]	Antibody: monoclonal anti-VEGF Scoring system: 0 = none, 1+ = < 5%, 2+ = 5–50%, 3+ = >50% positive tumor cells High reactivity cases had score above median value	Overexpression of VEGF had both unfavorable overall survival and disease-free survival

114 breast cancers [29]	Antibody: monoclonal anti-VEGF165 Scoring system: staining intensity was graded from 0 (no staining) to III (most intense staining)	VEGF had no prognostic significance for disease-free survival or cancer survival

100 invasive ductal carcinomas, NOS, 19 invasive lobular carcinomas [30]	Antibody: polyclonal anti-VEGF-C Staining was graded as strong, medium, or weak expression	VEGF-C had no prognostic significance for overall survival or disease-free survival

323 invasive breast carcinomas [31]	Antibody: monoclonal anti-VEGF Scoring system: sum of staining intensity (0 = negative to 3 = strong) and percentage of positive cells (0 = 0%, 1 = 1–25%, 2 = 26–50%, 3 = >50%) Positive cases had score 4–6	VEGF was not associated with incidence of relapse or death

181 invasive ductal carcinomas, 22 invasive lobular carcinoma, 8 invasive ductal and lobular (mixed) carcinomas, 5 ductal *in situ* carcinomas, 1 medullary carcinoma [32]	Antibody: anti-VEGF-C Scoring system: cytoplasmic staining was graded negative (negative), 1+ (10–39%), 2+ (40–69%), 3+ (>70%)	VEGF-C had no prognostic significance for disease-free survival

238 invasive breast cancers not receiving tamoxifen from 500 primary breast cancers using tissue microarrays [22]	Antibody: polyclonal anti-VEGF Scoring system: cytoplasmic staining intensity was scored from 0 to 3 High staining intensity group had score 3	VEGF had no prognostic significance for relapse-free survival

87 primary breast cancers [33]	Antibody: polyclonal anti-VEGF-C Scoring system: 0 (no staining or cytoplasmic staining in <10%), 1+ (faint cytoplasmic staining in >10%), 2+ (weak-to-moderate complete cytoplasmic staining in >10%), 3+ (strong complete cytoplasmic staining in >10%) Positive cases had score 2+ or 3+	VEGF-C had no prognostic significance for disease-free survival or overall survival

224 invasive breast cancers using tissue microarrays [44]	Antibody: polyclonal anti-VEGF Scoring system: staining intensity was graded from 0 (negative) to 3 (intense intensity), and the percentage of positive cells was recorded (0 = 0%, 1 = <1%, 2 = 1–10%, 3 = 10–50%, 4 = 50–90%, 5 = >90%) Positive cases are those having any positive staining	VEGF-A-positive cases had favorable disease-free survival at 10-year followup in multivariate analysis

207 invasive breast carcinomas [34]	Antibody: polyclonal anti-VEGF-D Scoring system: 0 = negative, 1 = weak focal staining, 2 strong focal/widespread moderate staining, 3 = strong widespread staining Positive cases had score 2 or 3	VEGF-D had no prognostic significance for overall survival or relapse-free survival

96 invasive ductal carcinomas, 9 other invasive carcinomas [45]	Antibody: monoclonal anti-VEGF-D Scoring system: positive cases had more than 10% tumor cells with cytoplasmic staining	(1) Positive VEGF-D cases had poorer disease-free survival in univariate and multivariate analyses (2) Positive VEGF-D cases had poorer overall survival in univariate analysis

228 invasive unilateral breast carcinomas [35]	Antibody: monoclonal anti-VEGF (isoforms 121, 165 and 189) Scoring system: positive cases had more than 1% immunoreactive tumor cells	VEGF had no prognostic significance for overall survival or relapse-free survival

114 invasive ductal carcinomas, 9 other invasive carcinomas [46]	Antibody: polyclonal anti-VEGF-C Scoring system: positive cases had more than 10% immunoreactive tumor cells	Positive VEGF-C cases had poorer disease-free survival and overall survival in univariate analysis
99 invasive ductal carcinomas, NOS [36]	Antibody: anti-VEGF Scoring system: positive cases had more than 10% tumor cells with membrane or cytoplasmic staining	VEGF had no prognostic significance for overall survival or relapse-free state

107 primary invasive breast carcinomas [47]	Antibody: anti-VEGF-A, anti-VEGF-C, anti-VEGF-D Scoring system: computer-assisted image analysis based on the percentage of immunostained surfaces and mean optical density High-expression group had value equal to or higher than median	(1) High-VEGF-A-expression cases had worser disease-free survival (2) VEGF-C or VEGF-D had no prognostic significance (3) Cases with both low VEGF-A and VEGF-C expression had better disease-free survival

242 node-negative breast cancer [37]	Antibody: polyclonal anti-VEGF isoforms 121, 165, 189, and 206 Scoring system: high-expression cases had >40% immunopositive tumor cells	VEGF had no prognostic significance for disease-free survival or overall survival

94 invasive breast cancer, 4 noninvasive cancer [48]	Antibody: polyclonal anti-VEGF-C Scoring system: positive cases had over 10% tumor cells stained positively	VEGF-C-positive group had poorer disease-free survival

**Table 2:** Summary of clinical studies exploring the link between tumor-associated macrophages and other clinicopathological parameters in invasive breast carcinomas.

Tumor type	Means of tumor-associated macrophages assessment	Findings
101 invasive breast carcinomas [7]	Macrophage marker: CD68 Macrophage index was determined by 25-point Chalkey graticule as the mean of three “hot spot” counts under 250x field	(1) High macrophage index correlated with high vascular grade (2) High macrophage index in poorly vascularized areas (3) High macrophage index predicted reduced relapse-free and overall survival

75 invasive breast carcinomas with lymphoplasmacytic infiltrates [67]	Macrophage marker: CD11c Macrophage was counted as percentage of total leukocyte infiltrate identified by CD45	(1) Macrophage predominance in leukocyte infiltrate correlated with high grade and c-erbB-2 expression

75 invasive breast carcinomas (50 ductal, 9 lobular, 5 mixed, 5 tubular/cribriform, 1 mucinous) [68]	Inflammation was classified as diffuse, perivascular, and perilobular on H&E and also using markers. Intensity was qualitatively graded as from 0 (absent) to 3 (marked) Macrophage marker: CD68	(1) In diffuse inflammation pattern, macrophage intensity predominated other cell types and was associated with high-grade, large tumor size, tumor necrosis, and c-erbB-2 expression (2) Intensity of diffuse inflammation but not macrophage correlated with vascularity

120 invasive breast carcinomas (60% ductal, 20% lobular, 20% others) [6]	Macrophage marker: CD68 (KP-1 antibody) Macrophages were counted in 40 hpf (20 hpf tumor cell zones and 20 hpf stromal zones) and graded from weak (<300) to intense (>500)	(1) Intensity of macrophage was higher in node-negative tumors (2) Intratumoral macrophage infiltration correlated with high tumor grade, absence of ER, and high mitotic grade

57 invasive breast carcinomas NOS (abstract) [69]	Macrophage marker: CD68	(1) Tumor-associated macrophages correlated with mitotic activity index

109 invasive breast carcinomas (ductal 88, lobular 8, others 13) [70]	Macrophage marker: CD68 Macrophage index was determined by 25-point Chalkey graticule as the mean of three “hot spot” counts under 250x field	(1) Higher macrophage index associated with necrosis

26 invasive ductal carcinomas (13 cases <5 years, 13 cases >5 years' survival) [71]	Macrophage marker: CD68 Hot spots were identified under 100x, field and macrophages were counted in 5 hpf under 400x field	(1) Higher macrophage count in poor prognosis group

151 invasive ductal carcinomas [72]	Macrophage marker: CD68 Macrophages were counted in 5 hot spots, and the mean of the highest three was determined (per mm^2^)	(1) High macrophage count correlated with high levels of macrophage chemoattractant protein-1 and thymidine phosphorylase in breast cancer by ELISA (2) High level of macrophage chemoattractant protein-1 had worsened relapse-free survival

96 invasive breast carcinomas (78 ductal, 7 lobular, 11 others) [73]	Macrophage marker: CD68 Macrophage index was determined by 25-point Chalkey graticule as the mean of three “hot spot” counts under 250x field	(1) Macrophage index correlated with high VEGF and EGFR expression (2) In EGFR-negative cases, high VEGF correlated with increased macrophage index, high grade, presence of necrosis, and increased tumor p53 expression (3) No significant prognostic value of VEGF

24 invasive breast carcinomas (12 ductal, 12 lobular) [74]	Macrophage marker: CD68 Macrophage index was determined by 25-point Chalkey graticule or by absolute count as the mean of five VEGF positive areas under 200x field. In VEGF-negative areas, 5 most or least vascularized areas were chosen	(1) Macrophage count was higher in less vascularized areas

230 invasive ductal carcinomas [75]	Macrophage marker: CD68 macrophages were counted in 5 hot spots, and the mean of the highest three was determined (per mm^2^). Graded from 0 (<50/mm^2^) to 2 (>100mm^2^)	(1) High macrophage count showed a tendency of correlation with high level of tumoral macrophage chemoattractant protein-1 by immunohistochemistry (*P* = .089). (2) High level of tumoral macrophage chemoattractant protein-1 showed a tendency of correlation with high microvessel density grade (*P* = .087)
97 invasive ductal carcinomas [76]	Macrophage marker: CD68 Macrophages were semiqualitatively graded as 1 = no macrophages, 2 = small foci of macrophages, 3 = large foci of macrophages 4 = diffuse macrophages infiltration in tumor stroma	(1) Higher macrophage grade associated with higher VEGF expression, higher microvessel density, and higher mitotic activity index

249 invasive ductal carcinomas (abstract) [77]	Macrophage density was assessed as average density of three hot spots at a magnification of 400x	(1) Macrophage density significantly correlated with both the VEGF expression and MVD (2) Macrophage density was associated with the nuclear grade, estrogen receptor status, and MIB-1 count (3) Patients with a high macrophage density had a significantly worse disease-free survival prognosis than those with a low density

97 breast carcinomas [78]	Macrophage marker: CD68 Macrophages were semiqualitatively graded as 1 = no macrophages, 2 = small and large foci of macrophages, 3 = diffuse macrophages infiltration in tumor stroma	(1) Macrophage grade was not correlated with tumor chemoattractant protein-1

78 invasive breast carcinomas (48 ductal, 30 lobular) [79]	Macrophage marker: HAM56 antibody Macrophages were semiqualitatively graded as 1 = no macrophages, 2 = small foci of macrophages, 3 = large foci of macrophages, 4 = diffuse macrophages infiltration in tumor stroma	(1) Higher macrophages in invasive ductal carcinomas compared to invasive lobular carcinomas (2) In invasive ductal carcinomas, macrophage grade correlated with tumor size, lymph node metastasis, stage, microvessel density, VEGF, and tumor grade (3) In invasive ductal carcinomas, macrophage grade and clinical stage were predictive in disease-free survival rate

133 invasive breast carcinomas (94 ductal, 28 lobular, 8 mucinous, 3 papillary) [80]	Macrophage marker: CD68 Macrophages were counted in 5 consecutive 400x fields in areas identified as “hot spots” under 100x	(1) Higher macrophage count associated with high tumor grade, p53 expression, absence of ER, high VEGF expression in macrophage, and postsurgical serum VEGF level

168 invasive primary breast cancer (142 ductal, 20 lobular, 6 others) [81]	Macrophage marker: CD68 Macrophages were counted using point counting method (expressed as percentage of volume occupied by a component out of total volume) using a 100-point ocular grid counting at 400x field over 30 fields and were grouped tertiles	(1) High tertile percentage of macrophage correlated with high tumor grade, high Ki-67 labelling, absence of hormonal receptors, high microvessel density, high CD4 and CD8 count

128 invasive ductal carcinomas [82]	Macrophage marker: CD68 Macrophages were counted as mean of the 3 densest areas at 200x field (per mm^2^) following a brief scan at low power and separated into <320 or >320/mm^2^ groups	(1) Macrophage count correlated with stromal chemoattractant protein-1 (2) Stromal chemoattractant protein-1 correlated with lymphatic invasion and predicted worsened relapse-free survival
